# Virtual escape rooms for therapeutic communication in nursing education

**DOI:** 10.1097/01.NURSE.0000854004.25753.10

**Published:** 2022-08-25

**Authors:** Sharon W. Hutchinson, Charlotte Hurst

**Affiliations:** At Dillard University's College of Nursing in New Orleans, La., **Sharon Hutchinson** is the dean and a professor of nursing, and **Charlotte Hurst** is a professor of nursing and the coordinator of the RN-to-BSN Program.

Therapeutic communication is an essential and fundamental component of nursing curricula and practice. It is a process where the nurse consciously influences or helps the patient achieve a better understanding through verbal and nonverbal communication.^1^ Some therapeutic communication strategies include active listening, acknowledging the patient's involvement, and seeking clarification. Nurses' behavioral responses that convey acceptance, respect, and sincerity to the patient are also considered therapeutic communication strategies.^2^ The goal of therapeutic communication is to establish a trusting relationship between the nurse and patient that facilitates cooperation, respect, and collaboration for decision-making, problem-solving, information sharing, and providing feedback, thereby enhancing the patient's physical and emotional well-being. Thus, educational content provided in remote learning environments needs to include therapeutic communication techniques.

Educational resources available to teach therapeutic communication remotely include simulations that accompany textbooks, video clips of actors, standardized patients, and digital standardized patient software.^3^-^6^ The latter, created by nursing textbook publishers, are replete with resources such as objectives, rubrics, and plan of care packets or concept maps that guide the student through the learning experience.^5^,^6^ However, the abrupt transition to remote learning due to the COVID-19 global pandemic has prompted the nursing faculty at a university in Louisiana to convert in-person teaching into a virtual experience: a virtual escape room to optimize simulation training in therapeutic communication for sophomore and junior nursing students.

## Virtual escape room simulation

While used in a variety of settings, an escape room is essentially a game wherein a group of players solves puzzles and challenges within a limited amount of time to achieve a specific goal—typically to get out of the room or game site.^7^ Game-based learning such as escape rooms have been used in educational environments.^7^

The nursing faculty designed a virtual version as a simulation activity based on the *Simulation Escape Room Workbook* that integrated the National Council State Board of Nursing Clinical Judgment Measurement Model's six cognitive skills for clinical judgment (NCJMM); the Quality and Safety Education for Nurses (QSEN) competencies; and the Situation, Background, Assessment, and Recommendation (SBAR) reporting model.^2^,^8^-^13^ The pilot for the virtual escape room was conducted in the Spring 2020 semester during the height of the pandemic and mandated stay-at-home order. It was offered in the following academic year.

The virtual escape room experience included an imaginary patient room and case scenarios to promote critical thinking and clinical judgment while using therapeutic communication. Students participated in teams of three and were given 3 hours to complete the challenges.

## Prebriefing

The virtual escape room simulation began with a prebriefing in which the faculty reviewed the purpose, objectives, and guidelines for the escape room. Prebriefing occurred in the main course room of the University's online learning management system and an open-access global teaching platform. The latter allowed the faculty to place students in smaller workrooms as they progressed through the simulation.

## Challenges

The virtual escape room experience began with a written SBAR report that was reviewed in the main virtual room/nurses station. At the end of the SBAR report, the student had to paraphrase the report to progress to the next breakout room. Students were then placed in breakout rooms/patient room, where they received clues and information on the case scenario, the change in the patient's health status, and a video link. To progress, the student had to ask patient-centered questions and determine the problem and the contributing factors. The challenges required the adequate use of therapeutic communication skills to identify the correct priority intervention for the patient.

**Table:** Simulation ranking

**Excellent = 5, Good = 4, Average = 3, Fair = 2, Poor = 1**
**Criteria/The participant was able to:**	**1**	**2**	**3**	**4**	**5**
Recognize relevant cues (QSEN: patient-centered care)					
Analyze cues to prioritize client needs (QSEN: patient-centered care)					
Demonstrate nurse's responsibilities in a non-threatening realistic online workplace environment (teamwork and collaboration)					
Communicate effectively with team members (oral and written; utilization of SBAR, teamwork and collaboration)					
Select appropriate equipment (safety)					
Utilize equipment correctly					

Note: For any rating less than average [3], provide feedback in the “comments” section.

The next breakout room addressed the process used to implement the intervention and the safe use of equipment. Upon completing these tasks, the faculty allowed the student to return to the main course room for debriefing.

## Debriefing

The faculty used a scripted debriefing guide to lead the students into the debriefing phase of the linear virtual escape room.

Scripted prompts facilitated the students' reflection to make further connections between course content and the experience, and to examine their feelings regarding the experience (see *Scripted prompts*). Faculty also used a Likert-type assessment tool based on the preidentified QSEN competencies (see *QSEN competencies*) to provide feedback regarding the student's performance as the student progressed through the linear escape room simulation (see *Simulation ranking*).

## Challenges

Challenges encountered included maintaining internet connectivity, ensuring that students had the appropriate technology at home, and ensuring that students were engaged and focused while in breakout rooms. Internet connectivity and technology access could not be addressed by the faculty. To compensate, the faculty extended the time of completion of the virtual escape room, and students were allowed to use cell phones in the absence of a computer. Breakout rooms were monitored, and voice access for faculty allowed them to serve as a bridge and refocus student learning. Time was also a challenge, as noted by students who wanted to complete the virtual escape room with no scheduled breaks. However, the faculty felt it important to schedule breaks to address possible fatigue and other physical needs.

## Conclusion

The COVID-19 pandemic created critical challenges in nursing education, particularly in teaching therapeutic communication via remote learning. Through a faculty-developed virtual linear escape room, students were able to engage in simulated patient-centered learning that addressed therapeutic communication. Faculty and student feedback indicate the experience was a positive learning activity. The virtual escape room experience is currently being reviewed by the College of Nursing Curriculum Committee as a mainstay student learning activity.

## Scripted prompts

- What are you feeling after this online activity?
- How did you feel when...?
- What were your strengths?
- What were your primary concerns in this scenario?
- What are the signs and symptoms the patient was exhibiting?
- Is there anything you would do differently next time?
- Let's identify a few take-home points that you will take away from this scenario and apply to your future practice.

## QSEN competencies^11^

- Patient-centered care
- Teamwork and collaboration
- Evidence-based practice
- Quality improvement
- Safety
- Informatics
